# Responsive mental health systems to address the poverty, homelessness and mental illness nexus: The Banyan experience from India

**DOI:** 10.1186/s13033-019-0313-8

**Published:** 2019-08-12

**Authors:** Lakshmi Narasimhan, Vandana Gopikumar, Vaishnavi Jayakumar, Joske Bunders, Barbara Regeer

**Affiliations:** 1 The Banyan, 6th Main Road, Mogappair West, Chennai, India; 2 The Banyan Academy of Leadership in Mental Health, Chennai, India; 3 Athena Institute for Research on Innovation and Communication in Health and Life Sciences, Faculty of Science, VU University Amsterdam, Amsterdam, The Netherlands; 4 School of Social Work, Tata Institute of Social Sciences, Mumbai, India

**Keywords:** Homelessness, Mental health, Health systems, User centred, Service development, Transition management

## Abstract

**Background:**

Mental health has gained prominence as a global public health priority. However, a substantial treatment gap persists in many low- and middle-income countries. Within this scenario, the nexus between homelessness, poverty and mental illness represents a particularly complex issue. This article presents the experience of The Banyan, a 25 years old non-profit organisation providing mental health care to people living in poverty in Tamil Nadu, India.

**Case presentation:**

The case study describes the evolution of The Banyan using a timeline narrative. By applying an action learning framework, the organisation’s evolution through four lifecycles, strategy and the key elements underlying mental health system responses are identified and presented. ‘User centred’ and ‘service integration’ emerge as the main dimensions of The Banyan’s responsive health system. Relating to these two attributes, a typology of services is derived, indicating the responsiveness of mental health systems in addressing complex problems. The role of the organisational culture and the expressed values during the transition is considered.

**Conclusions:**

The case study serves as an example of how responsive mental health systems may be constructed with both a user centred and a service integration focus.

## Background

Mental health has gained prominence as a global public health priority in the recent years. Mental disorders account for nearly 13% of the global disease burden according to one estimate [1], and are responsible for 37% of healthy years lost due to disease among non-communicable diseases [2]. Despite the development of evidence-based interventions, 75–90% people with mental illness in low- and medium-income countries (LMICs) are not in treatment [3]. Systemic barriers in LMICs such as lack of sufficient budgets and human resources, public health systems deficits, stigma and multidimensional poverty persist and prevent access to care.

India is home to an estimated 150 million people with mental illness [4], but only 10% of people with common mental disorders and only 40–50% of people with schizophrenia receive care [5]. Provision of mental health care in India, as in many other LMICs, faces a number of serious challenges. Mental health services are grossly inadequate [6] and tend to approach mental illness from a disease perspective [7], ignoring complex economic and social problems that contribute to wellbeing. In this context, the nexus between mental illness, poverty and homelessness represents a particularly persistent and complex problem [8]. Homelessness, poverty and mental ill-health are recursively related phenomena explained by both social causation and social drift [9]—people experiencing social and economic adversities have higher prevalence of and risks for mental ill-health [10–14], while those with mental health issues lapse into downward trajectories of poverty and homelessness [15–17]. In the Indian context, with sizeable scarcity of resources and services for mental health, homeless people with mental illness have few alternatives besides continuing on the streets or institutionalisation in mental health facilities [18] or beggar’s homes [19].

Health system reforms to address the treatment gap often focus on increasing availability of services using financial and human resources investments. While such reforms may increase capacity of services, the fundamental nature of the health system may remain unaltered. Given the complexity of the needs of the people who face the double jeopardy of homelessness and mental illness, more fundamental change is needed. Some civil society initiatives such as Ashadeep (Guwahati), Iswar Sankalp (Kolkata), Koshish (Mumbai), and state-run institutions such as the Hospital for Mental Health (Ahmedabad) and the Institute for Health and Behavioural Sciences (Delhi) have developed services to address the needs of homeless people with mental illness in India. Codifying such local responses and experiences can help generate socially robust knowledge and advance evidence-based practice and policy [20]. The primary aim of this article is to understand and describe the development of mental health system responses that may address needs of people with mental illness living in poverty and homelessness. To do this, the article presents a case study of the evolution of The Banyan, a non-profit organisation providing mental health care in India and reflects on implications for the nature of mental health systems. The secondary aim is to understand the organisational culture that promotes mental health system responses as a basis for scaling up such systems.

## Case presentation

The Banyan provides comprehensive mental health services in institutional and community settings for people experiencing poverty and homelessness in the states of Tamil Nadu, Kerala and Maharashtra. Starting in 1993 with a crisis intervention and rehabilitation centre for homeless women with mental illness in the city of Chennai, The Banyan’s continuum of care currently has three major services: Emergency Care and Recovery Services, Inclusive Living Options and NALAM: Community Mental Health Programme.

The Emergency Care and Recovery Services are primarily offered at a 120-bed facility for homeless women with mental illness in the city of Chennai. The services include crisis intervention, multi-disciplinary care, reintegration and aftercare interventions that aim to support users in finding pathways back to their families and communities of choice. The Emergency Care and Recovery Centre (ECRC) has been accessed by 1942 homeless women with mental illness, of whom 1478 (nearly three-quarters) have journeyed successfully back to their families all over India. About 40% of those in residential care participate in work, full-time or part time employment or social enterprises housed on campus. A 2010 study of a sample of women reintegrated from ECRC found that 20% were in paid employment and 61% were engaged in household occupational roles [21]. The same study found that 73.3% did not experience homelessness again and 84.4% remained in continued care through outpatient services. The ECRC approach has been adopted by the Government of Tamil Nadu. Five centres financed by the state are collaboratively run in District Hospitals. The reintegration component has been replicated collaboratively with the Government of Kerala across state-run psychiatric hospitals to help reunite long-stay clients with their families and access aftercare services.

Inclusive Living Options offer choice-based housing (congregate or non-congregate) with personalised supportive services (across work, socialisation, economic transactions, daily living and leisure), particularly for those who are unable to return to their families or live independently and are at risk of long-term institutionalisation in psychiatric facilities. About 200 people with mental illness with long-term care needs ranging from low to high disability levels are living in homes as part of formed families in rural and urban neighbourhoods with onsite support and case management at Clustered Group Homes (CGH) and Home Again (HA). A prospective 18-month evaluation between 2014 and 2016 of 53 participants of HA with matched controls in Care as Usual (CAU), the institutional facility, found a significant effect on community integration, which increased among participants in HA. Disability reduced significantly with time among participants of HA [22]. Similar results were found in a prospective evaluation using two-group design of 113 participants assigned to HA compared with the matched controls in CAU across sites in Tamil Nadu, Assam and Kerala. The intervention is being replicated with the Governments of Kerala and Maharashtra to address long-term institutionalisation in state mental hospitals.

NALAM (Tamil for wellness) is a community mental health programme that offers packages of care delivered by grassroots mobilisers supported through clinics co-located with primary health centres or community centres across rural and urban geographies. About 10,000 people have accessed proximal, comprehensive and personalised mental health care through NALAM. The services include outpatient and inpatient clinical care, home-based services, social entitlement facilitation, livelihood interventions, education and housing support, support groups and mental health promotion. Data from a cross-sectional survey of 346 women who accessed outpatient services between September 2015–December 2015 show that half perform independent occupational roles and a quarter of them are in paid employment. The Banyan has recently entered a collaboration with the Government of Tamil Nadu in two districts to strengthen the state-run District Mental Health Programme (DMHP) using the community engagement components of NALAM.

In order to address deficits in human resources, The Banyan Academy of Leadership in Mental Health (BALM) engages in education and research. BALM offers masters level programmes in Social Work and Applied Psychology (with specialisations in clinical psychology and counselling psychology) and a Diploma in Community Mental Health. Since inception, 229 students have graduated from the masters programmes and 116 have completed the Diploma. About three-quarters have continued to engage in the mental health sector, majorly in employment, with some in higher studies.

### Methods

For this case study, the authors retrospectively and qualitatively examine evolution of The Banyan’s responses in the mental health sector. Two qualitative methods were used: a timeline narrative of the organisation and analysis using action learning framework. A timeline narrative was constructed based on data from key informants, annual reports and evaluation reports. Insights from four key-informants were collected and integrated. Two of the four key informants were service users of The Banyan with over two decades of engagement. Both women were 45 years old at the time of the interview and were working at BALM. Two others were staff, one man and one woman, currently in senior management positions. The former has been with the organisation since inception and the latter since 2002. They were aged 45 and 29 years respectively at the time of the interview.

The evolution of The Banyan has been described in several documents such as annual reports and evaluation studies. Twenty annual reports (years 1993–2014) and three evaluation studies were examined retrospectively and qualitatively, covering critical changes and key elements of organisational strategy, in order to develop a conceptual framework for mental health system responses in the context of complex problems. A timeline narrative was constructed by LN and VG. Next, LN and BR coded the timeline narrative separately and analysed the coded data together using models of action reflection learning [23] in which knowledge is co-created by various participants who act and reflect on real world issues. This was reviewed independently by VG and JB, who sent feedback to LN, who then incorporated changes that were concordant and moderated discussions for changes that were discordant. This process was repeated until the four authors (VG, LN, JB and BR) agreed on the final output. Interpreter triangulation was facilitated through a recursive process of organising data individually and collectively reflecting and discussing the data. The four domains in action reflection learning processes—planning, action, observation, reflection [24]—were used to construct an analysis matrix:

- Planning: elements of narrative that constitute a strategic direction towards achieving organisational vision;
- Action: elements of narrative that involve activities to execute the plan;
- Observation: elements of narrative relating to what the organisation experienced following implementation of planning; and
- Reflection: narrative elements that express learning as result of the execution experience and the measurement of progress in congruence with the organisational vision.

### Lifecycles

Organisation of The Banyan’s narrative into the four domains of planning, action, observation and reflection revealed four distinct lifecycles into which the organic evolution of The Banyan may be categorised, each one with a plan originating from the reflections of the previous lifecycle. The Banyan started as a shelter service for homeless women with mental illness. The experiences of the co-founders in the months preceding the establishment, included public apathy to visible distress of a woman ‘in the middle of heavy traffic’ on a busy road in Chennai, the lack of any kind of facilities, and repeated encounters with many such women in distress.

The shelter facility started to provide a safe space for homeless women with mental illness and developed into a transit recovery space. With recovery of users and their expressed needs of living with family, The Banyan began facilitating reunion with families (Lifecycle 1). The positive response of many families and communities in welcoming back these women altered the organisation’s understanding of what had led to homelessness. The organisation also recognised that the magnitude of issue was far greater than initially anticipated; and that continuity of care post reintegration was critical.

From 1996 onwards, The Banyan expanded capacity by constructing new premises and began to offer multiple aftercare options for those who had left the shelter (Lifecycle 2). Reintegration became systematic and included options for self-discharge, employment, living in group homes and referrals to non-mental health institutions. As numbers continued to increase, The Banyan realised the importance of putting the needs of the constituency on the agendas of local and national governments. This involved an increasing emphasis on the role of the state and rights of homeless persons. In the provision of aftercare, The Banyan began to understand the difficulties of recovery in families living in poverty. Local care, expressed as a need by users, was hypothesised by a third party evaluation [25] as being important to maintaining recovery. In addition to challenges in providing aftercare, The Banyan experienced problems with institutional care: many people with long term needs were unable to exit the system and large communal spaces were not conducive to providing quality care.

During 2004–2012, user demands for alternate living spaces for those who could not return to their families led The Banyan to develop shared housing and community living options (Lifecycle 3). The need for continuity of care led to piloting of socioeconomic interventions (disability allowances, employment, housing support). Through this engagement in social care, The Banyan began to develop a deeper understanding of the perpetuating nature of inequities, including poverty, gender and old age. Local care did not always translate to benefits on the equity front, with poverty continually placing persons with mental illness at the risk of downward social drift and homelessness. The Banyan also learnt from user evaluations that positive outcomes are rooted in experiencing a better life. This led The Banyan to link care adherence to benefits that may mitigate socio-economic distress. The Banyan also began to collaborate with other stakeholders, such as the state mental health facility and other NGOs, to replicate its recovery and reintegration model. The challenges of providing institutional care rooted in an ethos of user self-determination became more evident: while there needed to be protocols for minimum services and processes, the key challenge was privileging user needs and rights among human resources. This led to the identification of human resources deficits in the mental health sector, not just in terms of numbers but also the lack of appropriate core values, leadership and multi-disciplinary intervention skills. Reflections on these developments led to changes in service integration during Lifecycle 4 from 2012 onwards, placing greater emphasis on recovery in the context of poverty. Greater understanding of the complex causal pathways to homelessness and of user expectations led to the adoption of a stronger development agenda within The Banyan, involving a comprehensive social care system of welfare entitlements and interventions targeting well-being. Quality assurance systems were introduced within institutional care, focusing on privacy and dignity during bathing, and the availability of a minimum level of assets such as fitted, coordinated clothes.

The organisation further integrated a well-being approach towards mental health into its community mental health programs by initiating the NALAM (Tamil for ‘wellness’) project. The project utilises village level wellness mobilisers to deliver a range of interventions from counselling to social welfare facilitation to promote outcomes in socio-economic spheres as a preventive strategy towards better mental health. The Banyan also investigated alternate service contact options for homeless persons with open shelters and street engagement in partnership with the Corporation of Chennai. Through this initiative the organisation has been able to offer flexible and user-initiated access options for homeless persons. Based on the NALAM approach, an active engagement with the community in vicinity, Dooming Kuppam, through life skills, skills development and other social interventions have been incorporated as a key component of this project.

In addition, driven by the success of independent living through shared housing in the villages and its impact on the lives of women who were once homeless, the organisation extended this approach towards addressing the needs of those who require higher support. This approach called Home Again, involves the facilitation of housing with graded levels of support, with a focus on facilitating socio-economic-political participation of users in communities.

The organisation recognised that there was a paucity in human resources aligned with the ethos and skills necessary to deliver interventions such as the ECRC, NALAM and Home Again. Therefore, masters level courses and diploma programmes were developed to cultivate critical perspectives and values by offering opportunities to learn by observing and doing in the real world.

Table 1 provides a summary of system level transitions in The Banyan’s mental health system. Action learning and the four domains of plan, act, observe, reflect constitute the main basis of the analysis in this discussion. The analysis matrix has been condensed into the table above to provide a summary of organisation level transitions in The Banyan’s mental health system. Narrative elements from plan and action have been combined and summarised under row labelled ‘Focus’; while those pertaining to ‘observation’ and ‘reflection’ are summarised under row labelled ‘Reasons for focus’ in Table 1. ‘Organisation level evolution’ based on the strategic shifts in ‘Focus’ of The Banyan have been demarcated into four lifecycles represented across the columns.

**Table 1:** Transitions in The Banyan’s strategy

Dimensions of Strategy	Lifecycle 1 1993–1996	Lifecycle 2 1996–2003	Lifecycle 3 2004–2012	Lifecycle 4 2012–present
Focus (planning and action)	Humanistic response to homeless women with mental illness: crisis intervention, shelter, reintegration, after-care	Institutional development: strengthening capacity, content and quality of responses	Mental health services in the community and stakeholder expansion, research and advocacy	Development of reconstructive approaches in community and institutional care with a focus on well-being: identity/personal growth/self-determination/meaning in life Building capacities among human resources to integrate theory and field practice
Reasons for focus (observation and reflection)	Lack of adequate responses to address deprivation and violence faced by homeless women with mental illness	Scale of issue in quantity as well as complexity; Infrastructure development with strengthening of systems of care for efficiency perceived as solution	Lack of access to care and continuity in care hypothesised to stem from presence vs absence deficits in community mental health Model formulation and dissemination of protocols as a means of scaling up	Micro and complex issues: non-linearity in the relationship between poverty, mental health, access to care and outcomes Need for development focus to mental health services integration Ethos/Core values scaling as opposed to standard operating protocols given dynamic nature of issue
Description of constituency	Homeless women with mental illness	Homeless women with mental illness and their families and communities	Persons with mental illness and their families living in poverty and homelessness in urban and rural areas	Persons, families and communities with psychosocial concerns living in poverty and homelessness in low resource settings Human resources in development and mental health sector
Description of stakeholder system	Hospitals, media, general public, philanthropists, social clubs	Other civil society organisations, hospitals, media, corporates, general public, government	Government, Other civil society organisations, hospitals, media, corporates, general public	Academic community, international institutions, local governments, federated and unfederated community institutions, Governments, Other civil society organisations, Hospitals, Media, Corporates, General Public

The analysis shows that over two decades of work in homelessness and mental health, The Banyan’s focus has developed from crisis intervention for homeless women with mental illness to an *integrated service* approach, including a wide variety of responses to clients’ psychological, financial, cultural and social well-being. Indicative of the change from crisis intervention to prevention to supporting well-being is the growing constituency that is considered service-user of The Banyan’s, including communities in low-resource settings at large.

In parallel, and as a response to the need for developing human resources in new directions, the stakeholder system that developed around The Banyan expanded to include non-traditional partnerships that are instrumental to The Banyan’s success. During The Banyan’s evolution, it became evident that focusing on one element of the problem was not enough: clients are caught in a multidimensional trap that needs to be approached in a tailor-made, *user*-*centred* way. Both micro and organisation level transitions in The Banyan have been based on increasing understanding of user demands and needs. Clients have evolved from service users to participants to owners. For example, homeless women with mental illness who once used The Banyan’s shelter services now work as mental health ‘change agents’ in their communities. Initially prioritising user self-determination (Lifecycle 1), the organisation engaged in an ongoing dialogue with its service users to gauge their experiences, needs and outcome definitions. This process of dialogue for co-creating knowledge served as the basis for formulating systemic responses. As a consequence of the user centred focus, The Banyan worked towards service integration in terms of quantitatively enhancing service diversity and levels, and qualitatively enhancing levels of integration within the delivery system. Responses to emerging user input, such as a trip to reunite with family (Lifecycle 1 to 2) and alleviating financial distress (Lifecycle 2 to 3), were institutionalised as systematic mechanisms, a process that increased comprehensiveness of both horizontal and vertical service offerings. Dialogue with users helped The Banyan understand that intersecting influences of social disadvantage and trauma on mental health, rather than untreated symptoms, are implicated in the urban reality of homelessness among those with mental illness [26]. This informed the organisation’s evolution from crisis intervention and shelter in family-like settings to integrated health and social care services on a continuum that expanded to include community mental health and community living options.

Thus, two key attributes emerge from the analysis of The Banyan’s evolution: ‘user centred’, placing the user at the centre in defining responses; and ‘service integration’, namely instituting appropriate, multiple responses at an organisational level. To understand how these developments and organisational health systems can be scaled up, we consider the organisational culture driving this process of development.

### Organisational culture and values

Organisational culture is like an iceberg, only a small part of it is visible or manifest [27]. Expressed values represent the key aspect of manifest culture [27] and, for that reason, values consistently apparent throughout the organisation’s evolution represent the focus of this analysis of organisational culture.

Although there have been considerable changes in The Banyan’s strategic focus since 1993, the expressed values have remained unchanged: commitment to the well-being of people, drive to understand the needs of clients, acceptance of the complexity of the reality of clients, and willingness to reconsider the identity of the organisation. These values have been built into the organisational culture through a number of processes:

- listening to the needs of clients during an ongoing dialogue in which all staff members participate;^1^
- maintaining high staff morale under extremely difficult circumstance by articulating, sharing and celebrating achievements, large and small;
- achieving synergy between ambitions, competences and activities by actively dialoguing with all network partners and staff members;
- combining in-depth understanding of the problem with the bigger picture by stimulating continuous reflection among stakeholders

**Fig. 1:**
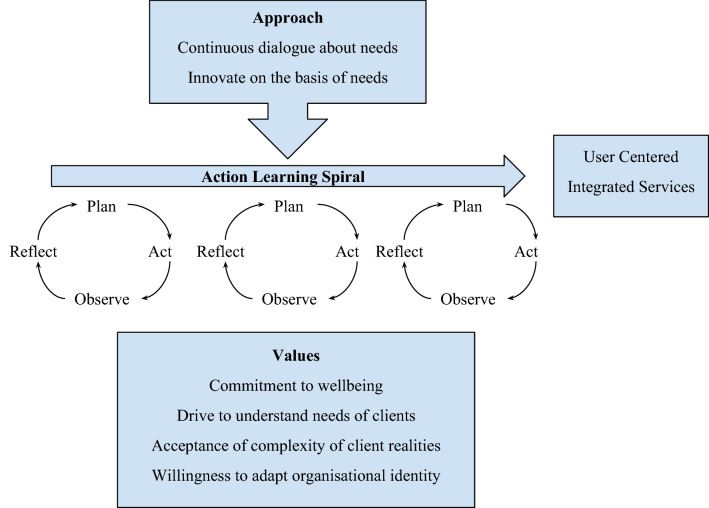
Value driven Action Learning Spiral (Authors)

These values help to promote an organisational culture that is anchored in an action learning approach, which is centred on a continuous dialogue with clients leading to innovation in practices, services and structures. While the transitions in The Banyan’s evolution emerge from one lifecycle to another, they were preceded by a process of co-developing these services at a micro level with service users. This dynamic nature of change driven by the constituency is a key feature, wherein responses are ongoing leading up to organisation level change, rather than in discrete blocks of planning, acting and evaluating (Fig. 1).

Several challenges accompany the evolution of The Banyan. These include: the paucity of resources, provision of aftercare to distributed geographies with scare local mental health services, disconnect between health and social welfare systems, and the exclusion of mental health and homelessness from a wide array of social entitlements.

From a systems development perspective, two important lessons may inform initiatives in mental health that may take a similar path. First, collaborative reflections among users and staff that combine quantitative indicators along with qualitative lived experience and practice narratives can assist in the process of evolving systems. The Banyan’s data have been organised to feed into quantifiable indicators tracked by a monitoring and evaluation system only in the last 6 years, whereas these may have been useful to inform strategies from inception. For instance, in the initial years, the institutional facility grew exponentially in an attempt to cater to the unmet needs of homeless women with mental illness. During this period, the proportion of long-stay users in the facility grew, duration of stay before discharge increased and maintaining quality standards became challenging. ECRC’s bed-strength was subsequently fixed at 120 and long-stay options were developed. Second, diffusion of values and new practices organisation wide (across programmes and staff) may call for continuous processes of immersive-learning and supervision, beyond didactic dissemination and trainings.

## Discussion

### Typology of mental health system responses

**Fig. 2:**
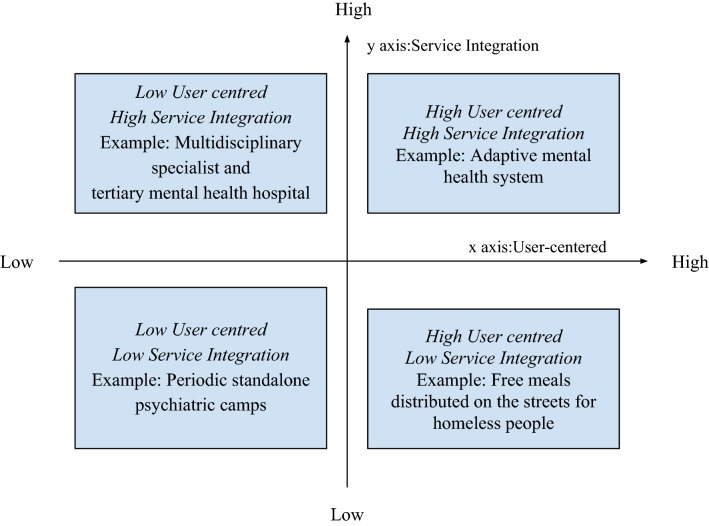
Typology of mental health system responses

Based on the dimensions ‘service integration’ and ‘user centred’ emerging from analysis of The Banyan’s mental health system, we introduce a typology of mental health system responses (Fig. 2) which may be used as a heuristic tool to understand differences between types of mental health services.

The typology presents four ideal-types, which by definition, do not correspond one-on-one to empirically observed phenomena. Rather, an ideal-type, as coined by Max Weber [28] represents an analytical construct based on specifically accentuated characteristics of a concrete phenomenon. They are used to understand, rather than represent social reality. At the same time, exemplars can be identified—phenomena that serve as an exceptional example of an analytical construct [29].

In this typology, the x-axis represents the degree to which services in a health system are ‘user centred’, while the y-axis represents the degree to which services in a health system demonstrates ‘service integration’. Responses of agents (individuals or organisations) within the health system may be highly user centred, but may not be integrated in a systematic manner. An example is a response that involves distribution of free meals to homeless persons on the streets. Some initiatives involve delivery of repackaged leftover food (from restaurants and other such establishments) or freshly cooked food by volunteers or staff to homeless people. They address a vital need, particularly for homeless people with mental illness, some of whom may not access soup kitchens or other such food sources. As a standalone service, while on a case-to-case basis other emerging needs such as health may be met, there is no process at a systems level, which may lead to limited scoping and outreach. On the other hand, one may also have highly integrated services, which are constructed without placing the user at the centre. An example may be multidisciplinary specialist tertiary mental health hospital with no processes for service user participation. Several disciplines such as psychiatry, psychology, social work and occupational therapy are involved in the process of delivering care, but such coordinated efforts may be driven by the expert perspectives of what user needs are. In such an instance, despite sophistication, services may continue to not appropriately address user needs. Unidimensional interventions that are again not changing with user input, such as periodic standalone psychiatric camps to screen, diagnose and dispense medication with no grassroots follow up mechanism, are another type of health system response. Such responses if fulfilling an aspect of user needs may potentially lead to impact that are restricted to shorter cycles, while failing to build these into long term gains. Finally, highly user centred and service integrated responses are those that adapt to emerging needs and priorities, change direction if needed and swiftly adopt innovative services.

### Scaling up: from organisational level to the health system

History shows the health system reform through top-down measures has been largely ineffective while bottom-up initiatives have often failed to scale up [30]. It is increasingly recognised that components of health systems should be perceived as interlinked and that they are a product of the social, economic, cultural and political context in which they are developed [31]. Taking such a complex adaptive system perspective to health systems has implications for health system reform. New approaches for system change have been developed and tested in the emerging research fields of system innovation [32, 33] and transition theory [34], originally developed in the fields of sustainable agriculture and energy, and more recently applied in the field of health care and health system reform [30]. It poses that the actions of agents at the micro level of a system are governed by laws, regulations, cultural values, beliefs of the meso level of a system, also referred to as the regime. It explains why new initiatives at micro level often fail to scale up to reform the system [35]; new practices are not adopted, or even counteracted, by incumbent institutions at regime level.

Increasingly it is argued that adaptive capacity is essential not only for the organisations that are part of a health system, but also for the health system as a whole [30, 31]. It is for instance argued that health systems should not only be seen in terms of its components (the people, institutions and resources that deliver health care services to meet the health needs of target populations) and their interrelationships, but also in terms of their ‘responsiveness to legitimate expectations’ [36]. Besides responding to users’ expectations, crossing boundaries (for example, between health and social welfare ministries, local and international organisations) is seen as a key attribute of mental health systems [31]. These scholars perceive health system reform beyond the development and diffusion of new interventions and increasing human capacity and instead argue for a move towards an adaptive (mental) health system that listens to needs (user centred) and responds by innovating practices through crossing boundaries and integrating services.

## Conclusions

This analysis of The Banyan’s evolution suggests a dynamic, responsive and system innovation approach to complex problems at the cusp of the poverty, homelessness and mental illness nexus. Mental health system formulations that are resonant with user systems, wherein services are personalised, organised and delivered around user characteristics and needs, may be more responsive to marginalised populations such as those living in homelessness and poverty. Ingrained in such responsive systems are value driven processes that systematically translate the knowledge acquired from the grassroots and subsequent innovations at the micro level into meso level institutions and macro level policy. This may be achieved through dialogue and synergy between various stakeholders while consistently reflecting on the bigger picture and pushing boundaries for what is aspired for change. Radical learning, that can alter the course of unresolved, complex and persistent problems, is made possible through a collaborative shared space for continuous reflection and action. Service delivery frameworks may require mechanisms that can continually translate the same into mental health formulations for practice. This may call for bottom up health systems, unique to the constituency they serve, with core value frameworks serving as the replicable components of such systems.

## Data Availability

Data and materials will be shared upon request to Lakshmi Narasimhan, The Banyan, India.

## Footnotes

1 The Banyan conducts an annual service user audit. There are meetings of user-carer groups and an external human rights committee monthly that serve to record and incorporate the voice of users into service design and delivery.
